# Authentication of *Zingiber* Species Based on Analysis of Metabolite Profiles

**DOI:** 10.3389/fpls.2021.705446

**Published:** 2021-11-22

**Authors:** Chenxi Wang, Yue Zhang, Hui Ding, Meifang Song, Jiaxin Yin, Heshui Yu, Zheng Li, Lifeng Han, Zhonglian Zhang

**Affiliations:** ^1^Tianjin State Key Laboratory of Component-Based Chinese Medicine, Tianjin Key Laboratory of TCM Chemistry and Analysis, Tianjin University of Traditional Chinese Medicine, Tianjin, China; ^2^Yunnan Key Laboratory of Southern Medicine Utilization, Yunnan Branch of Institute of Medicinal Plant Development, Chinese Academy of Medical Sciences, Peking Union Medical College, Jinghong, China

**Keywords:** *Zingiber*, chloroplast genome, untargeted metabolomics, gas chromatography-mass spectrometry, chemometric methods

## Abstract

*Zingiber corallinum* and *Zingiber montanum*, which belong to the Zingiberaceae family, are traditional Chinese folk medicinal herbs in Guizhou and Yunnan Province of China. They share great similarities in morphology, chemical constituent, and DNA barcoding sequence. The taxonomy of the two *Zingiber* species is controversial and discrimination of traditional Chinese medicines directly affects the pharmacological and clinical effects. In the present study, we performed a systemic analysis of “super-barcode” and untargeted metabolomics between *Z. corallinum* and *Z. montanum* using chloroplast (cp) genome sequencing and gas chromatography-mass spectrometry (GC-MS) analysis. Comparison and phylogenetic analysis of cp genomes of the two *Zingiber* species showed that the cp genome could not guarantee the accuracy of identification. An untargeted metabolomics strategy combining GC-MS with chemometric methods was proposed to distinguish the *Zingiber* samples of known variety. A total of 51 volatile compounds extracted from *Z. corallinum and Z. montanum* were identified, and nine compounds were selected as candidate metabolic markers to reveal the significant difference between *Z. corallinum and Z. montanum*. The performance of the untargeted metabolomic approach was verified with unknown *Zingiber* samples. Although the cp genomes could not be used to identify *Zingiber* species in this study, it will still provide a valuable genomics resource for population studies in the Zingiberaceae family, and the GC-MS based metabolic fingerprint is more promising for species identification and safe application of *Z. corallinum and Z. montanum*.

## Introduction

Traditional Dai Medicine (TDM), and recorded in the “Bei Ye Jing” 2,500 years ago, is one of the ancient ethnomedicine in China (Zhang et al., 2012). Bu Lei is one of the TDMs, and it is also a common folk medicinal material in Yunnan, Guizhou Province and other places of China. It has the effect of strengthening the stomach and eliminating accumulation. It has been used in traditional medicine for the treatment of food swelling, abdominal pain, nausea and vomiting, hepatosplenomegaly, and hot rheumatic pain (Sharifi-Rad et al., 2017; Brillatz et al., 2020). There are two species of this medicine, namely, the rhizome of *Zingiber corallinum* and *Zingiber montanum*. It is very difficult to distinguish them because of the quiet similarity of plant appearance, medicinal properties, and microstructure (Rafi et al., 2013). However, the discrimination of traditional Chinese medicines (TCMs) directly affects the pharmacological and clinical effect (Duan et al., 2011). Consequently, the discrimination of *Z. corallinum* and *Z. montanum* becomes a very important issue for patients.

Currently, identifying species in TCMs is changing more easily and scientifically. In recent years, the chloroplast (cp) genomes have shown great potential for species identification, especially between closely related herbal species (Daniell et al., 2016; Rendón-Anaya et al., 2017; Ma et al., 2019; Teske et al., 2020). Therefore, the development of the cp genome resources of the *Z. corallinum* and *Z. montanum* is not only conducive to the accurate identification of closely related species, but can also greatly promote the improvement of chloroplast genetic engineering (Kelly et al., 2011). Cp is an energy converter that can convert light energy into chemical energy and release oxygen through photosynthesis (Li et al., 2014). The cp genome is a naked circular double-stranded DNA molecule that consists of a small single copy (SSC), a large single copy (LSC), and two inverted repeats (IRa and IRb) (Li et al., 2015; Liang et al., 2019). Due to the conservation of its structure, length, and gene types, the cp genome has been proven to have the significance of species identification and has been widely used in cp genetic engineering such as genetic diversity analysis, species DNA molecular identification, and molecular phylogeny research (Li et al., 2014; Jin and Daniell, 2015). At present, the number of cp genome sequences deposited in the National Center for Biotechnology Information (NCBI) is dramatically increasing (Zhou et al., 2017). However, in terms of the types of the entire plant community, the total number of plants with the determined cp genome sequence is far from enough (Rawal et al., 2020). Therefore, the next step should be to add more plant cp genome data in order to provide scientific basis for species resource protection and pharmacognosy research.

Apart from the above method, mass spectrometry (MS) based metabolomics methods have been widely used in the identification of TCM species due to their high sensitivity and resolution (Masson et al., 2014; Wang et al., 2017; Zhang et al., 2018). Compared with the cp genome, the advantage of MS-based metabolomics is that there is no need to establish whole-genome sequencing, and the types of metabolites are much smaller than the number of genes, thus providing higher resolution (Duan et al., 2011; Endara et al., 2018; Teske et al., 2020; Lee et al., 2021). However, for TCMs with highly similar genetic relationships and new analytical techniques, it is inevitable to process a large amount of chemical measurement data. Therefore, it is essential to develop a chemometric strategy to deal with the huge information obtained from a mass analyzer (Wang et al., 2018; Liang et al., 2021; Pollo et al., 2021). Multivariate statistical analysis like principal component analysis (PCA), partial least squares discrimination analysis (PLS-DA), and orthogonal PLS-DA (OPLS-DA) have received more and more attention in the field of analyzing massive data (Black et al., 2016; Palmioli et al., 2020). Metabolomics methods based on multivariate statistical analysis have proven to be a powerful tool for phytochemical classification by finding differences in metabolites (Claassen et al., 2019; Ma et al., 2019; Zhang et al., 2020).

In this study, the phylogenetic relationships and chemical compositions between two *Zingiber* species were revealed to differentiate similar TCMs, in terms of genomics and untargeted metabolomics. Firstly, we conducted the complete cp genomes of *Z. corallinum* and *Z. montanum* sampled from Guizhou and Yunnan. However, it had not succeeded in distinguishing the two *Zingiber* species due to the high genetic similarity. Then, an untargeted metabolomic approach combining GC-MS was used to discriminate the *Zingiber* samples of known variety. Using multivariate statistical analysis, nine volatile compounds were selected as metabolic markers to distinguish the two herb materials. The reliability of the strategy was confirmed by identifying species of unknown samples. Our research is the first time to integrate cp genome sequencing technology combined with chemometric-based untargeted metabolomic method, which could be applied in the discrimination of the two *Zingiber* types and exploration of the metabolomics approach on discrimination and study of other TCMs.

## Materials and Methods

### Plant Materials and Chemicals

Plant materials from two *Zingiber* species were collected from their main producing areas. A total of 25 samples of *Z. corallinum* were collected from Bijie City, Guizhou Province; Tongren City, Guizhou Province and Xishuangbanna Banna Pharmaceutical Co., Ltd., Yunnan Province, respectively. Thirteen samples of *Z. montanum* were collected from Xishuangbanna Banna Pharmaceutical Co., Ltd. Additionally, 22 unknown samples were used to test the proposed strategy. Finally, the above herbs were authenticated by Professor Zhong-Lian Zhang from Yunnan Branch of Institute of Medicinal Plant Development, Chinese Academy of Medical Sciences. Their geographical origins are described in Supplementary Table 1 and Supplementary Figure 1.

Analytical grade petroleum ether with a boiling range of 60–90°C was purchased from Tianjin Concord Technology Co., Ltd. HPLC grade ethyl acetate was obtained from Thermo Fisher (NJ, United States).

C8-C20 n-alkanes standard solution was purchased from Sigma-Aldrich (St. Louis, MO, United States). Reference compounds of isovanillin, terpinene-4-ol, β-sesquiphellandrene, and (*E*)-4-(3,4-dimethoxyphenyl) but-3-en-1-ol were obtained from Shanghai Yuanye Biotech. Co., Ltd., (Shanghai, China).

### DNA Extraction

*Zingiber corallinum* and *Z. montanum* fresh leaves were collected from Yunnan Province. Clean leaves of samples frozen at −80°C using TaKaRa MiniBEST Universal Genomic DNA Extraction Kit with a standard protocol extract the total genomic DNA (TaKaRa, Beijing, Japan), and DNA quality was assessed using a Nanodrop 2000 (Thermo, United States). The OD260/280 values ranged from 1.8 to 2.0, and more than 2 μg of DNA was equally pooled from the two species individuals to generate shotgun libraries.

### Chloroplast Genome Sequencing, Assembly, and Annotation

The cpDNA samples were randomly fragmentation buffer and broken into 300–500 bp in Covaris M220 Focused-ultrasonicator (Covaris, Woburn, MA, United States). The A and B connector was connected at both ends of the DNA fragment. The segments were screened and then the self-connecting segments of the connector was removed. The library was sequenced using Illumina HiSeq4000 sequencing platform at the Major Company (Shanghai, China). PCR amplification was performed in eight cycles for library enrichment, and purpose strips were recovered by electrophoresis in 2% (w/v) Low Range Ultra Agarose. Bridge PCR amplification was performed on the cBot solid phase (Truseq PE Cluster Kit v3-cBot-HS) carrier to generate clusters. Then, two libraries were sequenced using Illumina HiSeq4000 sequencing platform to obtain 2 × 150 bp paired-end reads at the Biozeron Company (Shanghai, China).

A total of 6,763,299,900 and 8,310,381,900 bp raw data were generated with a paired end read length of 150 bp. Low-quality read data and the clean read for cp genome assembly was filtered out. The reference database was created by related species cp sequences downloaded from the NCBI. Then, the high-quality read was mapped to the database and the mapped reads were extracted on the basis of coverage and similarity. The extracted readings were assembled into a contig using SOAPdenovo2 (Luo et al., 2012) and the resulting contigs were combined and extended to obtain complete chloroplast genome sequence. Then, optimization of the assembly results by GapCloser software (v1.12) repairs the inner hole of the assembly result. Finally, the position of the LSC, SSC, and IR regions of the chloroplast genome were determined by localization, and complete chloroplast genome sequence including LSC, SSC, and two IR regions were obtained.

Annotation of the *Z. corallinum* and *Z. montanum* cp genomes were executed using the online program Dual Organellar GenoMe Annotator (DOGMA^1^) (Wyman et al., 2004), coupled with manual correction. The identification of tRNA genes were performed by software tRNAscan-SE (v2.0, University of California, Santa Cruz, CA, United States) (Schattner et al., 2005) and DOGMA (Tamura et al., 2013). The Organellar Genome DRAW (OGDRAW) (v1.2, Max Planck Institute of Molecular Plant Physiology, Potsdam, Germany) (Lohse et al., 2007) was used to draw the gene map with default settings. Finally generate cp genomes.sqn file were submitted to NCBI.

### Chloroplast Genome Structure, Codon Usage, and SSR Analysis

The CodonW software (University of Texas, Houston, TX, United States) with the relative synonymous codon usage (RSCU) ratio was used to investigate the distribution of codon (Sharp and Li, 1987). Conserved sequences were identified between the cp genomes of two *Zingiber* species using BLASTN with an E-value cutoff of 1e-10. The reference sequences were used for verifying boundaries genes, intron/exon, and coding regions. Moreover, The guanine and cytosine nucleobases (GC) content was analyzed using MEGA 6.0 (Tamura et al., 2013). Moreover, the software MISA^2^ was used to detect simple sequence repeats (SSRs), with parameters set to encompass the number of repeat units of a mononucleotide SSR higher than or equal to 10, followed by higher than or equal to five and four repeat units for di and trinucleotide SSRs, respectively, and higher than or equal to three repeat units for tetra, penta, and hexanucleotides, respectively.

### Genome Comparison Analysis and Phylogenetic Analysis

Nucleotide variability (Pi) in the cp genome of *Z. corallinum*, *Z. montanum*, and *Z. montanum* (MK262727) were observed by sliding window analysis using DnaSP (Rozas et al., 2017) software. The mVISTA (Frazer et al., 2004) program in Shuffle-LAGAN mode was employed to compare the cp genome of *Z. corallinum*, *Z. montanum*, and *Z. montanum* (MK262727). To determine phylogenetic positions of two *Zingiber* species within Zingiberaceae, 38 cp genomes were downloaded from NCBI to construct a phylogenetic tree. MAFFT (Katoh et al., 2005) was used for sequence alignment. Phylogenetic trees were reconstructed based on the 40 complete cp genome sequences by Neighbor-Joining (NJ) and Maximum Parsimony (MP) methods in the software MEGA 6.0 (Tamura et al., 2013) with 1,000 bootstrap replicates.

### Sample Preparation for GC-MS Analysis

The sliced rhizomes of *Z. corallinum* and *Z. montanum* were dried at 50°C, and the dried rhizomes were crushed into crude powder and then passed through a 50-mesh sieve. Three hundred twenty milliliters of petroleum ether as extraction solvent was added to a conical flask containing 40 g of the powder at a material-to-liquid ratio of 1:8 (g/ml). The essential oil was extracted from the powder during 5 and 1 h period using the heating reflux extraction method and ultrasonic extraction method, respectively. The extract was concentrated under reduced pressure to obtain volatile oil. The weight of volatile oil was then measured and the extraction yield was calculated. The oil was dissolved with an appropriate amount of ethyl acetate and then vortexed for 5 min. After centrifugation (Eppendorf 5424R, Barkhausenweg 1, Hamburg, Germany) at 13,200 *g* for 20 min. The supernatant was analyzed for GC-MS analysis.

### GC-MS Analysis

Gas chromatography was carried out on an Agilent 7890B GC system (Agilent Technologies, Palo Alto, CA, United States) equipped with an HP-5 fused silica column (30 m × 250 mm × 0.25 μm). The oven temperature was programmed at 40°C for 4 min and then increased to 75°C at a rate of 35°C/min, and finally increased 5°C/min to 250°C. Injector and detector temperatures were 250 and 250°C, respectively. The carrier gas was high-purity helium, and the injection volume was 1 μl.

Mass spectrometric analysis was performed using an Agilent 7000D GC/MS Triple Quad with an EI ion source. The ion source temperature was 230°C with an ionization energy of 70 eV, a scan time of 0.3 s and a mass range of 30–500 amu. The acceleration voltage was turned on after a solvent delay of 3.5 min.

The volatile components were identified by matching their retention indices (RI) relative to the C8-C20 n-alkanes standard and comparing the mass spectra of each component with the reference mass spectra *via* National Institute of Standards and Technology (NIST) database. The specific steps were as follows. Firstly, a match quality of 80% minimum was used as a criteria, and only the components with the mass spectrum fragment matching rate greater than 80% were used for the next analysis. Subsequently, the RI of the sample components corrected by C8-C20 n-alkanes was compared with the RI of the standard substance in the NIST library and the literature data, and the higher match was given priority to be identified.

### Metabolomics Approach With Multivariate Statistical Analysis

The first step is establishing of the model for discrimination of *Z. corallinum* and *Z. montanum*. The original data format was converted into mzXML by using ProteoWizard’s msConvert tool. Then spectra acquisition and spectral data pre-processing were implemented with R Project. After processing of normalization, PCA and OPLS-DA were applied to visualize the variance among *Z. corallinum* and *Z. montanum* by SIMCA 14.1 software (Umetrics, Sweden). R^2^X and R^2^Y, respectively, represent the percentage of X and Y matrix information explained by a model, while Q^2^Y is used to evaluate the predictive ability of the model. The closer R^2^X, R^2^Y, and Q^2^Y are to 1, the better the data fitting effect of the model will be. Based on the variable importance (VIP) values (VIP > 1.5) from an OPLS-DA model, a number of metabolites responsible for the differentiation of the metabolic profiles of species could be obtained. After the method was established, the verification set was then analyzed in order to estimate the predictive capability of the established method, to minimize the specific risk of overfitting.

## Results

### Chloroplast Genome Features of *Zingiber corallinum* and *Zingiber montanum*

**FIGURE 1:**
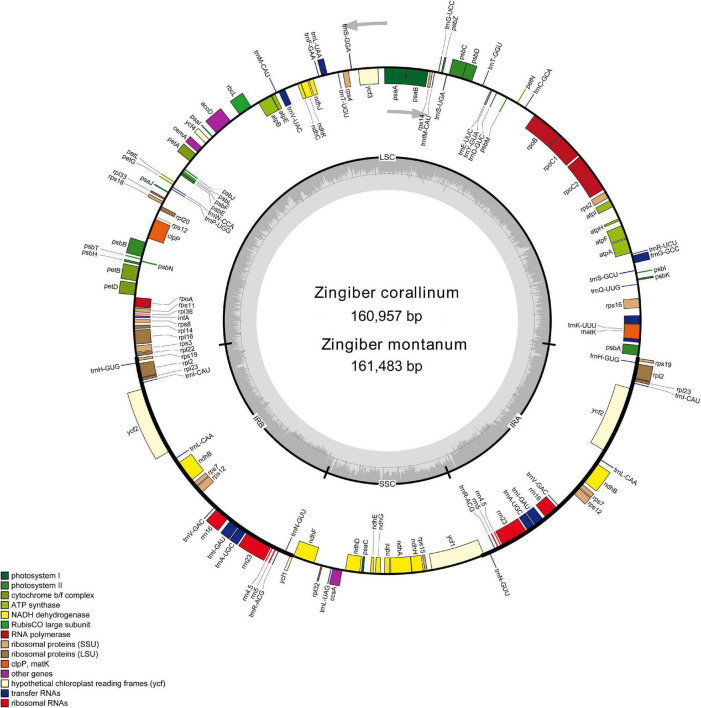
Gene map of the *Z. corallinum and Z. montanum* complete chloroplast genome. Genes that are inside and outside the circle are transcribed clockwise and counterclockwise, respectively. Genes belonging to different functional groups are classified by different colors. The darker gray area in the inner circle corresponds to GC content, whereas the lighter gray area corresponds to AT content.

Two complete cp genome sequences of *Z. corallinum* and *Z. montanum* were submitted to GenBank database, and their accession numbers were MW801385 and MW801386, respectively. The complete cp genomes of *Z. corallinum* and *Z. montanum* composed of a single circular molecule with obvious quadripartite structure (Figure 1). The length of *Z. corallinum* and *Z. montanum* cp genomes were 160,957 and 161,483 bp, including a large single-copy (LSC) region of 87,835 and 87,925 bp, a small single-copy (SSC) region of 19,488 and 19,488 bp, and a pair of inverted repeats (IRa and IRb) of 26,817 and 27,035 bp each. Moreover, the GC content of the genomes were 35.86 and 35.81% in *Z. corallinum* and *Z. montanum*, respectively. The distribution of GC content was different in different regions of the cp genome sequences. The GC content in the IR regions were the highest (41.9 and 41.7%) than that of the LSC regions (33.6%) and SSC regions (29.3%) (Table 1). The codon AT content of the two *Zingiber* species is the same, AT content at the third codon position (71.1%) was higher than that at the second (62.5%) and first (55.2%) positions in the protein-coding genes. Additionally, the AT content was the highest (70.7%) in the SSC region, followed by the LSC region (66.4%), and the lowest (58.2 and 58.3%) in the IR regions (Supplementary Table 2).

**TABLE 1:** The general features of *Z. corallinum* and *Z. montanum* chloroplast genomes.

Genome characteristics	*Z. corallinum*	*Z. montanum*
GenBank number	MW801385	MW801386
Genome size (bp)	160,957	161,483
Total genes	132	132
CDS	86	86
tRNA genes	38	38
rRNA genes	8	8
GC content (%)	35.86	35.81
**LSC**		
Length (bp)	87,835	87,925
GC content (%)	33.6	33.6
**SSC**		
Length (bp)	19,488	19,488
GC content (%)	29.3	29.3
**IR**		
Length (bp)	26,817	27,035
GC content (%)	41.9	41.7

A total of 133 different genes were identified from *Z. corallinum* and *Z. montanum* cp genome, including 87 protein-coding genes, eight rRNA genes, and 38 distinct tRNA genes (Table 2). The distribution of genes in the two cp genomes was exactly the same. Among these genes, a total of 20 genes (eight protein-coding genes, eight tRNA genes, and four rRNAs) were duplicated in the IR regions. The LSC region included 60 protein-coding genes and 21 tRNA genes. The SSC regions contained 11 protein-coding genes and one tRNA gene. The ycf1 gene spans the SSC and IRa region (Table 2, Supplementary Table 3, and Figure 1). There was no difference in the number and location of genes except the length of LSC and IR.

**TABLE 2:** Genes present in the chloroplast genomes of two *Zingiber* species.

Group of genes	Gene names	Amount
Photosystem I	*psaA*, *psaB*, *psaC*, *psaI*, *psaJ*	5
Photosystem II	*psbA*, *psbB*, *psbC*, *psbD*, *psbE*, *psbF*, *psbH*, *psbI*, *psbJ*, *psbK*, *psbL*, *psbM*, *psbN*, *psbT*, *psbZ*	15
Cytochrome b/f complex	*petA*, petB*, petD*, *petG*, *petL*, *petN*	6
ATP synthase	*atpA*, *atpB*, *atpE*, atpF*, *atpH*, *atpI*	6
NADH dehydrogenase	ndhA*, ndhB* (× 2), *ndhC*, *ndhD*, *ndhE*, *ndhF*, *ndhG*, *ndhH*, *ndhI*, *ndhJ*, *ndhK*	12
RubisCO large subunit	*rbcL*	1
RNA polymerase	*rpoA*, *rpoB*, rpoC1*, *rpoC2*	4
Ribosomal proteins (SSU)	*rps2*, *rps3*, *rps4*, *rps7* (× 2), *rps8*, *rps11*, rps12* (× 2), *rps14*, *rps15*, rps16*, *rps18*, *rps19* (× 2)	15
Ribosomal proteins (LSU)	rpl2* (× 2), *rpl14*, rpl16*, *rpl20*, *rpl22*, *rpl23* (× 2), *rpl32*, *rpl33*, *rpl36*	11
Other genes	*accD*, clpP**, *matK*, *ccsA*, *cemA*, *infA*	6
Proteins of unknown function	*ycf1* (× 2), *ycf2* (× 2), ycf3**, *ycf4*	6
Transfer RNAs	38 *tRNA*s (six contain an intron, eight in the IRs)	38
Ribosomal RNAs	*rrn4.5* (× 2), *rrn5* (× 2), *rrn16* (× 2), *rrn23* (× 2)	8

*Gene containing one intron; **gene containing two introns; (× 2) gene with two copies.

### Codon Usage and Simple Sequence Repeats

Relative Synonymous Codon Usage (RSCU) is the ratio of the usage frequency of a particular codon to the expected frequency, which can reflect the usage of synonymous codon in the coding sequences. RSCU = 1 means that the codons have no bias. The frequency of codons usage are lower than the expected RSCU value < 1.00, and RSCU > 1.00 indicate use of a codon more frequently than expected (Sharp and Li, 1987). In this study, we analyzed the codon usage frequency of *Z. corallinum* and *Z. montanum* cp genome based on RSCU. As shown in Supplementary Figure 2 and Supplementary Table 4, the codon number and frequency of *Z. corallinum* and *Z. montanum* were identical. The cp protein-coding genes of these two species contained 61 codons encoding 20 amino acids. Eighty-seven protein-coding genes comprised 26,488 codons in both the *Z. corallinum* and *Z. montanum*. Among these codons, those for arginine (AGA), leucine (UUA), and alanine (GCU) were the most common in both *Z. corallinum* and *Z. montanum* chloroplast genomes. Meanwhile, we found that thirty codons showed the codon usage bias (RSCU > 1) in the cp genes of two *Zingiber* species. Other than leucine (UUG), amino acid codons in two *Zingiber* species cp genomes of preferentially ended with A or U (RSCU > 1), and codons ending with A or U accounted for 71.1%. The start codon AUG and UGG encoding methionine and tryptophan were not biased (RSCU = 1).

Simple sequence repeats (SSRs) are tandemly repeated sequences widely distributed in chloroplast genomes, are composed of 1–6 nucleotide repeating units, and are usually used as an important molecular marker for species identification and diversity analysis (Powell et al., 1995). Here, we analyzed the SSRs contained in the cp genomes of *Z. corallinum* and *Z. montanum*, including the distribution and types. According to Table 3, the distribution and types of SSRs in *Z. corallinum* and *Z. montanum* were exactly the same. A total of 110 SSRs were identified using the microsatellite identification tool (MISA) from the cp genomes of two *Zingiber* species. The most abundant type was repeated dinucleotide (36.36%), followed by mononucleotide (34.55%), tetranucleotide (16.36%), trinucleotide (9.10%), pentanucleotide (2.73%), and hexanucleotide repeats (0.90%). Most of these repeats were located in the LSC region with 78 repeats, the IRs region with the least repeats (10), and the SSC region with 22 repeats. Among these SSRs, the AT/TA dinucleotide repeats were the most abundant motif, followed by A/T mononucleotide repeat, and then AAAT/ATTT tetranucleotide repeats. The codon number and frequency, SSRs location and types of *Z. corallinum* and *Z. montanum* were identical.

**TABLE 3:** The simple sequence repeat (SSR) types of the two chloroplast genomes of *Zingiber* species.

SSR Type	Repeat Unit	Amount	
		*Z. corallinum*	*Z. montanum*
Mono	A/T	37	37
	C/G	1	1
Di	AG/CT	2	2
	AT/TA	38	38
Tri	AAG/CTT	2	2
	ACT/AGT	0	0
	AGG/CCT	0	0
	AAT/ATT	8	8
Tetra	AAAC/GTTT	1	1
	AAAG/CTTT	2	2
	AAAT/ATTT	12	12
	AACT/AGTT	2	2
	AATG/ATTC	1	1
	AATT/AATT	0	0
	ACAT/ATGT	0	0
Penta	AAAAT/ATTTT	1	1
	AATAT/ATATT	1	1
	AAGGT/ACCTT	0	0
	AAATT/AATTT	0	0
	AACAT/ATGTT	0	0

### Comparative Genome Analysis

**FIGURE 2:**
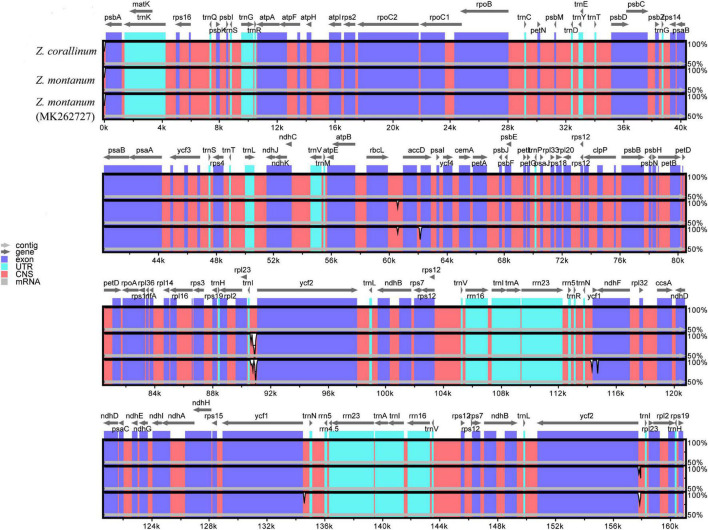
Sequence alignment of three Zingiber chloroplast sequences with *Z. corallinum* as reference by using in mVISTA. The Y-scale represents the percentage of identity ranging from 50 to 100%.

From the above analysis, we found that the cp genomes of *Z. corallinum* and *Z. montanum* had very little difference. We used DnaSP (Rozas et al., 2017) software to determine the polymorphic sites and found that two cp genomes had no variable sites, and *Z. corallinum* had deletion fragments 526 bp relative to *Z. montanum*. Meanwhile, we downloaded the *Z. montanum* (MK262727) sequence published by NCBI (Li et al., 2020a). To determine the level of sequence similarity and find potential difference sites between the two *Zingiber* species, we compared and analyzed the sequence homology of *Z. corallinum*, *Z. montanum*, and *Z. montanum* (MK262727) using the mVISTA software, with *Z. corallinum* being used as reference sequence (Figure 2). The comparison showed that the differences among the three sequences were very small, and the only difference sites were found located in the non-coding regions. The difference between *Z. corallinum* and *Z. montanum* existed within intergenic spacers (IGS): rbcL-accD, trnI-CAU-ycf2. The difference regions of *Z. corallinum*, *Z. montanum*, and *Z. montanum* (MK262727) were located in accD, trnN-GUU-ycf1 and ycf1-ndhF.

Furthermore, variable regions in the cp genome of *Z. corallinum*, *Z. montanum*, and *Z. montanum* (MK262727) were observed by sliding window analysis using DnaSP software. The results showed that the variable regions were located in trnI-CAU-ycf2, ycf1-ndhF, and ndhF, and their nucleotide variability (Pi) values were 0.0033 and 0.0044, respectively (Supplementary Figure 3). As a whole, the cp genome sequence variation of two *Zingiber* species was very small. There was no highly variable region that can be used as potential DNA marker in the cp genome of the two *Zingiber* species.

### Phylogenetic Analysis

**FIGURE 3:**
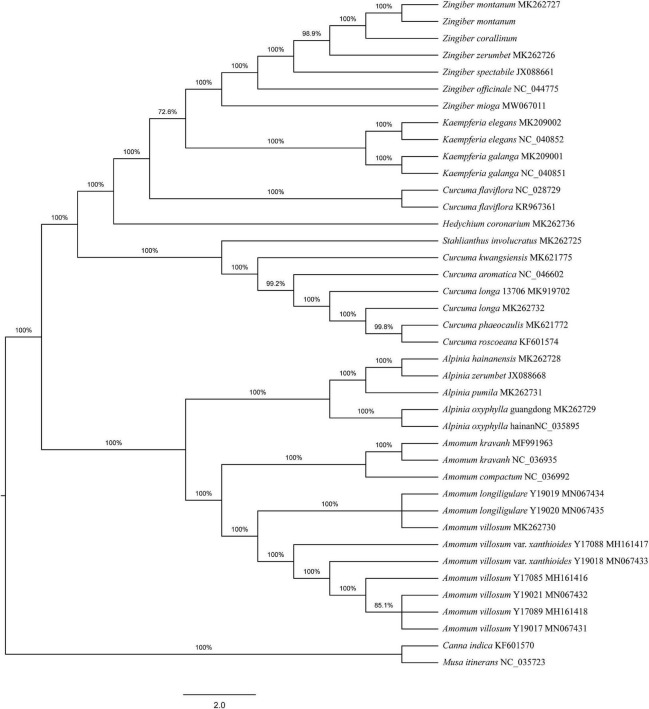
Phylogenetic tree constructed using Neighbor-Joining (NJ) based on complete cp genomes of 36 Zingiberaceae species. Numbers above the branches are the bootstrap support values.

To determine the phylogenetic positions of the two *Zingiber* species within the Zingiberaceae, 36 complete cp genome sequences belonging to seven genera of Zingiberaceae (including five sequences each from *Zingiber* and *Alpinia*, four sequences from *Kaempferia*, 12 sequences from *Amomum*, eight sequences from *Curcuma*, and one sequence each from *Stahlianthus* and *Hedychium*), *Canna indica*, and *Musa itinerans* from Musaceae as outgroups were obtained from NCBI. Both the MP and NJ phylogenetic trees indicated that the six species of the genus *Zingiber* were clustered together and separated from the other six genera of the family Zingiberaceae (Supplementary Figure 4 and Figure 3). The NJ tree confirmed the *Z. montanum* and *Z. montanum* (MK262727) into a branch (support values = 100%), and *Z. corallinum* exhibited a sister relationship. But the MP tree confirmed the *Z. montanum*, *Z. montanum* (MK262727), and *Z. corallinum* into a branch (support values = 100%). This outcome indicated that the sequences difference between *Z. corallinum* and *Z. montanum* were very infinitely small, and the cp genome could not guarantee accurate identification. Meanwhile, two phylogeny trees both showed that the two genera *Alpinia* and *Amomum* as sister groups into a branch were strongly supported (support values ≥ 95%).

### Identification of Chemical Composition in Two *Zingiber* Species

The yields of the volatile oil by heating reflux extraction method and ultrasonic extraction method were 9.52 and 8.67% (*Z. corallinum*), and 16.89 and 15.14% (*Z. montanum*), respectively. We finally used the ultrasonic extraction method to extract the volatile oil due to its simple operation and shorter time, although the ultrasonic extraction method had a slightly lower yield of the volatile oil. The GC-MS analysis of the rhizome of two *Zingiber* species exhibited total ion (TIC) chromatograms as shown in Supplementary Figure 5. As a result, a total of 46 common compounds were identified, in which four thereof were positively identified by comparing with standards, and the remaining 42 were tentatively identified by matching the retention indices and mass spectra with NIST database and the literature data (Jiang et al., 2006; Yang et al., 2009; Kiran et al., 2013; Pang et al., 2020; Pintatum et al., 2020). The volatile constituents present in the two species separately were presented in Tables 4, 5 using a percentage of peak area. Based on the compounds that were identified from the two species samples, we found that there was only a difference in content level between these two species, but no apparent composition difference in their GC-MS chromatograms.

**TABLE 4:** The Characterization of volatile oil ingredients in *Z. corallinum* by gas chromatography-mass spectrometry (GC-MS).

No.	RI_cal_ (iu)	RI_NIST_ (iu)	Formula	Identification	Peak area percentage (%)
1	937.9	937.0	C_10_H_16_	α-Pinene	0.30
2	980.6	974.0	C_10_H_16_	Sabinene	6.50
3	994.8	991.0	C_10_H_16_	β-Myrcene	0.49
4	1,021.3	1,017.0	C_10_H_16_	α-Terpinene	0.68
5	1,029.9	1,025.0	C_10_H_14_	*p*-Cymene	0.39
6	1,034.3	1,037.0	C_10_H_16_	β-Ocimene	0.68
7	1,064.5	1,060.0	C_10_H_16_	γ-Terpinene	1.42
8	1,073.6	1,075.0	C_10_H_18_O	4-Thujanol	0.39
9	1,093.6	1,088.0	C_10_H_16_	α-Terpinolene	0.29
10	1,104.6	1,106.6	C_10_H_18_O	*trans*-4-Thujanol	0.50
11	1,128.4	1,130.2	C_10_H_18_O	*cis*-*p*-Menth-2-en-1-ol	0.23
12	1,147.1	1,150.4	C_10_H_18_O	*trans*-*p*-Menth-2-en-1-ol	0.21
13	1,183.7	1,177.0	C_10_H_18_O	Terpinene-4-ol	7.18
14	1,197.7	1,189.0	C_10_H_18_O	*p*-Menth-1-en-8-ol	0.20
15	1,202.3	1,204.0	C_10_H_18_O	*trans*-*p*-Menth-1-en-3-ol	0.09
16	1,225.7	1,217.6	C_10_H_18_O	γ-Terpineol	0.09
17	1,260.3	1,256.4	C_12_H_20_O_2_	γ-Terpineol acetate	0.20
18	1,277.4	1,270.4	C_10_H_18_O_2_	*trans*-*p*-Menth-2-ene-1,4-diol	0.03
19	1,293.0	1,286.9	C_12_H_20_O_2_	2-Camphanol acetate	0.09
20	1,298.1	1,295.0	C_10_H_14_O	*p*-cymene-7-ol	0.02
21	1,306.2	1,301.0	C_12_H_20_O_2_	Terpinene 4-acetate	0.13
22	1,357.0	1,355.5	C_12_H_20_O_2_	*p*-Menth-1-en-8-ol, acetate	0.42
23	1,398.1	1,391.0	C_15_H_24_	β-Elemen	0.04
24	1,408.4	1,401.0	C_8_H_8_O_3_	Isovanillin	0.10
25	1,441.5	1,433.0	C_15_H_24_	γ-Elemene	0.19
26	1,462.7	1,468.5	C_15_H_24_	(*E*)-β-Farnesene	0.14
27	1,476.9	1,469.7	C_11_H_16_O_2_	2-Methoxy-4-butylphenol	0.03
28	1,486.3	1,478.9	C_11_H_14_O_2_	(*Z*)-4-(But-1-en-1-yl) guaiacol	0.44
29	1,491.2	1,495.0	C_9_H_10_O_3_	Benzaldehyde, 3,4-dimethoxy-	0.45
30	1,501.0	1,497.3	C_15_H_24_	α-Zingiberene	0.38
31	1,515.4	1,514.4	C_15_H_24_	β-Bisabolene	0.24
32	1,531.2	1,526.4	C_15_H_24_	β-Sesquiphellandrene	4.15
33	1,546.8	1,522.0	C_12_H_16_O_2_	(*Z*)-4-(but-1-en-1-yl)-1,2-dimethoxybenzene	0.03
34	1,557.3	1,549.4	C_11_H_14_O_2_	(*E*)-4-(But-1-en-1-yl) guaiacol	0.29
35	1,578.6	1,570.0	C_12_H_14_O_2_	Benzene, 4-(1*Z*)-1,3-butadien-1-yl-1,2-dimethoxy-	0.19
36	1,603.4	1,595.0	C_12_H_16_O_2_	(*E*)-4-(But-1-en-1-yl)-1,2-dimethoxybenzene	2.47
37	1,640.2	1,636.0	C_12_H_14_O_2_	Benzene, 4-(1*E*)-1,3-butadien-1-yl-1,2-dimethoxy-	23.4
38	1,642.5	1,641.0	C_12_H_14_O_2_	Triquinacene, 1,4-bis(methoxy)	0.21
39	1,692.5	1,688.3	C_12_H_16_O_3_	Asarone	0.04
40	1,695.8	1,686.0	C_13_H_18_O_3_	(*Z*)-1-(2,4,5-Trimethoxyphenyl) but-1-ene	0.16
41	1,749.1	1,738.3	C_13_H_16_O_3_	(*Z*)-1-(Buta-1,3-dien-1-yl)-2,4,5-trimethoxybenzene	0.17
42	1,780.0	1,770.0	C_13_H_18_O_3_	(*E*)-1-(2,4,5-Trimethoxyphenyl) but-1-ene	1.46
43	1,794.0	1,791.0	C_11_H_12_O_3_	2-Propenal, 3-(3,4-dimethoxyphenyl)-	0.59
44	1,839.4	1,827.0	C_13_H_16_O_3_	Triquinacene, 1,4,7-tris(methoxy)-	11.54
45	1,884.1	1,869.0	C_12_H_16_O_3_	(*E*)-4-(3,4-Dimethoxyphenyl) but-3-en-1-ol	11.55
46	1,998.0	1,994.0	C_14_H_18_O_4_	(*E*)-4-(3,4-Dimethoxyphenyl) but-3-en-1-yl acetate	0.42

*RI_Cal._, experimentally determined; RI_NIST_, retention indices from NIST database.*

**TABLE 5:** The Characterization of volatile oil ingredients in *Z. montanum* by GC-MS.

No.	RI_cal_ (iu)	RI_NIST_ (iu)	Formula	Identification	Peak area percentage (%)
1	937.6	937.0	C_10_H_16_	α-Pinene	0.48
2	981.1	974.0	C_10_H_16_	Sabinene	8.06
3	994.8	991.0	C_10_H_16_	β-Myrcene	0.80
4	1,021.3	1,017.0	C_10_H_16_	α-Terpinene	1.05
5	1,030.2	1,025.0	C_10_H_14_	*p*-Cymene	0.87
6	1,034.6	1,037.0	C_10_H_16_	β-Ocimene	1.45
7	1,064.5	1,060.0	C_10_H_16_	γ-Terpinene	2.12
8	1,073.6	1,075.0	C_10_H_18_O	4-Thujanol	0.44
9	1,093.6	1,088.0	C_10_H_16_	α-Terpinolene	0.52
10	1,104.6	1,106.6	C_10_H_18_O	*trans*-4-Thujanol	0.56
11	1,128.4	1,130.2	C_10_H_18_O	*cis*-*p*-Menth-2-en-1-ol	0.23
12	1,147.1	1,150.4	C_10_H_18_O	*trans*-*p*-Menth-2-en-1-ol	0.21
13	1,183.7	1,177.0	C_10_H_18_O	Terpinene-4-ol	8.89
14	1,197.7	1,189.0	C_10_H_18_O	*p*-Menth-1-en-8-ol	0.25
15	1,202.3	1,204.0	C_10_H_18_O	*trans*-*p*-Menth-1-en-3-ol	0.11
16	1,225.7	1,217.6	C_10_H_18_O	γ-Terpineol	0.06
17	1,260.3	1,256.4	C_12_H_20_O_2_	γ-Terpineol acetate	0.10
18	1,277.4	1,270.4	C_10_H_18_O_2_	*trans*-*p*-Menth-2-ene-1,4-diol	0.03
19	1,292.6	1,286.9	C_12_H_20_O_2_	2-Camphanol acetate	0.13
20	1,297.9	1,295.0	C_10_H_14_O	*p*-cymene-7-ol	0.04
21	1,306.2	1,301.0	C_12_H_20_O_2_	Terpinene 4-acetate	0.04
22	1,357.0	1,355.5	C_12_H_20_O_2_	*p*-Menth-1-en-8-ol, acetate	0.53
23	1,398.1	1,391.0	C_15_H_24_	β-Elemen	0.05
24	1,408.4	1,401.0	C_8_H_8_O_3_	Isovanillin	0.46
25	1,441.5	1,433.0	C_15_H_24_	γ-Elemene	0.32
26	1,462.6	1,468.5	C_15_H_24_	(*E*)-β-Farnesene	0.08
27	1,476.9	1,469.7	C_11_H_16_O_2_	2-Methoxy-4-butylphenol	0.06
28	1,486.2	1,478.9	C_11_H_14_O_2_	(*Z*)-4-(But-1-en-1-yl) guaiacol	0.38
29	1,491.3	1,495.0	C_9_H_10_O_3_	Benzaldehyde, 3,4-dimethoxy-	0.35
30	1,501.2	1,497.3	C_15_H_24_	α-Zingiberene	0.64
31	1,515.4	1,514.4	C_15_H_24_	β-Bisabolene	0.38
32	1,533.5	1,526.4	C_15_H_24_	β-Sesquiphellandrene	3.27
33	1,546.8	1,522.0	C_12_H_16_O_2_	(*Z*)-4-(but-1-en-1-yl)-1,2-dimethoxybenzene	0.02
34	1,557.1	1,549.4	C_11_H_14_O_2_	(*E*)-4-(But-1-en-1-yl) guaiacol	0.24
35	1,578.6	1,570.0	C_12_H_14_O_2_	Benzene, 4-(1*Z*)-1,3-butadien-1-yl-1,2-dimethoxy-	0.22
36	1,603.6	1,595.0	C_12_H_16_O_2_	(*E*)-4-(But-1-en-1-yl)-1,2-dimethoxybenzene	3.27
37	1,640.2	1,636.0	C_12_H_14_O_2_	Benzene, 4-(1*E*)-1,3-butadien-1-yl-1,2-dimethoxy-	29.95
38	1,646.4	1,641.0	C_12_H_14_O_2_	Triquinacene, 1,4-bis(methoxy)	0.11
39	1,692.5	1,688.3	C_12_H_16_O_3_	Asarone	0.05
40	1,695.9	1,686.0	C_13_H_18_O_3_	(*Z*)-1-(2,4,5-Trimethoxyphenyl) but-1-ene	0.21
41	1,748.9	1,738.3	C_13_H_16_O_3_	(*Z*)-1-(Buta-1,3-dien-1-yl)-2,4,5-trimethoxybenzene	0.14
42	1,780.0	1,770.0	C_13_H_18_O_3_	(*E*)-1-(2,4,5-Trimethoxyphenyl) but-1-ene	2.09
43	1,791.9	1,791.0	C_11_H_12_O_3_	2-Propenal, 3-(3,4-dimethoxyphenyl)-	0.36
44	1,836.1	1,827.0	C_13_H_16_O_3_	Triquinacene, 1,4,7-tris(methoxy)-	8.94
45	1,884.4	1,869.0	C_12_H_16_O_3_	(*E*)-4-(3,4-Dimethoxyphenyl) but-3-en-1-ol	6.52
46	2,000.0	1,994.0	C_14_H_18_O_4_	(*E*)-4-(3,4-Dimethoxyphenyl) but-3-en-1-yl acetate	0.13

*RI_Cal._, experimentally determined; RI_NIST_: retention indices from NIST database.*

### Metabolomic Analysis for Discrimination of *Zingiber corallinum* and *Zingiber montanum*

**FIGURE 4:**
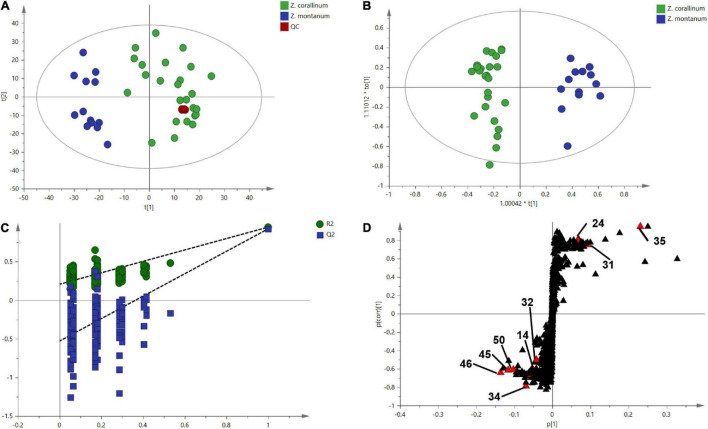
Multivariate statistical analysis of the training set from gas chromatography-mass spectrometry (GC-MS). The green, blue and red circles represent *Z. corallinum*, *Z. montanum*, and QC samples: **(A)** PCA score plot, the x-axis and y-axis, respectively, represented the correlation coefficient between the variance and the first two principle components (t1 and t2) which accounted for 26.1 and 19.7% of total variance, respectively; **(B)** Orthogonal partial least squares discrimination analysis (OPLS-DA) scores plots of *Z. corallinum* vs. *Z. montanum*, the x-axis and y-axis represented the weights of the regression coefficients of the predicted principal components and orthogonal principal components, respectively; **(C)** 200-permutation test of OPLS-DA models, the x-axis represented the accuracy of the model, and the y-axis represented the frequency of the accuracy of the 200-permutation test; **(D)** S-plot diagram comparing the *Z. corallinum* and *Z. montanum* groups, S-plot provides visualization of the OPLS-DA predictive component loading to facilitate model interpretation.

**FIGURE 5:**
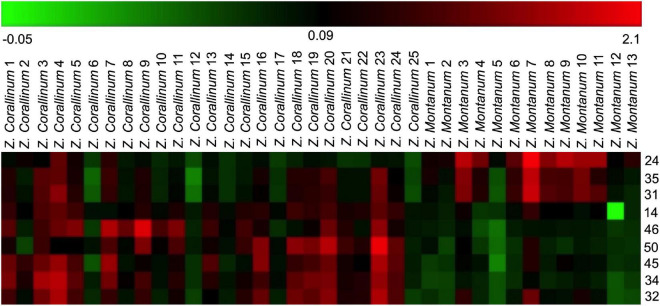
Heatmap of identified distinguished metabolites of *Z. corallinum* and *Z. montanum* by the samples of known variety. Red and green colors indicate an increase and a decrease of metabolite level, respectively.

One thousand one hundred sixty-eight MS ion markers were extracted from the GC-MS dataset, and a PCA was performed with them to obtain a preliminary overview of general similarities and differences between samples. PCA score plot showed the 38 samples were clearly separated into *Z. corallinum* (marked by a green dot) and *Z. montanum* (marked by a blue dot) with its first two components in Figure 4A. The two *Zingiber* Species could be separated clearly by a supervised OPLS-DA (Figure 4B). The R^2^X, R^2^Y, and Q^2^ of this model were 0.747, 0.947, and 0.924, respectively. The 200-permutation test (R^2^ intercept 0.214 and Q^2^ intercept −0.504) validated the stability and reliability of this OPLS-DA model (Figure 4C). Subsequently, relying on the two criteria-VIP value of OPLS-DA model (VIP > 1.5) and *p* value of *T* test (*p* < 0.05), nine metabolites could be presumably considered as candidate biomarkers. At the same time, by comparing the *Z. corallinum* with *Z. montanum* group, S-plot of the OPLS-DA model showed that a total of nine metabolites were labeled to be the main contributors (Figure 4D). In order to visualize the difference in the distribution of metabolites, a heat map was plotted by MeV (Multiple Experiment Viewer) 4.9.0 software (Figure 5). High expression levels exhibit red color, and low expression levels exhibit green. Among these metabolites, the concentration of isovanillin* (24), benzene, 4-(1*E*)-1,3-butadien-1-yl-1,2-dimethoxy- (35) and (*E*)-4-(but-1-en-1-yl)-1,2-dimethoxybenzene (31) showed accumulated effects, while (*Z*)-4-(but-1-en-1-yl)-1,2-dimethoxybenzene (34), (*E*)-4-(3,4-dimethoxyphenyl)but-3-en-1-ol* (45), triquinacene, 1,4-bis(methoxy) (46), triquinacene,1,4,7-tris(methoxy)- (50), terpinene-4-ol* (14), and β-sesquiphellandrene* (32) decreased compared to the *Z. corallinum* samples. (*: means the compounds were compared with standards).

### Confirmation of the Reliability of the Metabolomic Approach

**FIGURE 6:**
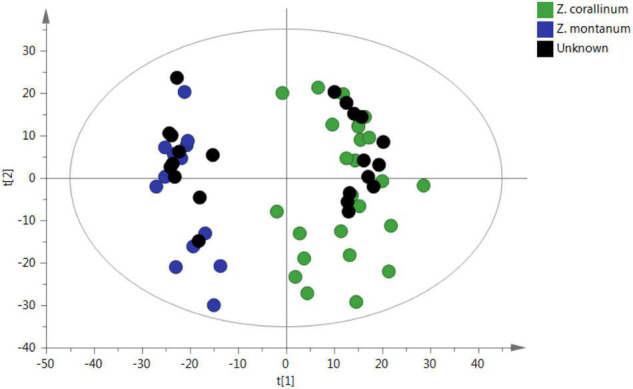
PCA prediction of unknown samples from GC-MS. The black circle represents the unknown sample. [The x-axis and y-axis, respectively, represented the correlation coefficient between the variance and the first two principle components (t1 and t2)].

To confirm the reliability of the untargeted metabolomic approach in distinguishing *Z. corallinum* and *Z. montanum*, 22 unknown samples were submitted to the same analytical procedure as the whole set of previous samples. Then, unknown samples were statistically analyzed *via* PCA in order to prove that an unsupervised method will classify these unknown samples within the different groups. As a result, all 22 unknown samples were assigned into the correct cluster within the PCA (Figure 6). Furthermore, the abundance profiles of nine key metabolites of the unknown 1–12 samples were found to be very similar to those of *Z. corallinum* samples, whereas the profiles of the unknown 13–22 samples exhibited similar trend compared to the *Z. montanum* samples (Supplementary Figure 6). These results were confirmed to be reliable by the authentication of 22 unknown samples by Professor Zhong-Lian Zhang from Yunnan Branch of Institute of Medicinal Plant Development, Chinese Academy of Medical Sciences. Therefore, the untargeted metabolomics strategy could unequivocally classify the species of unknown samples.

## Discussion

*Zingiber corallinum* and *Z. montanum* have a highly similarity in external morphology, medicinal properties, and microstructure, so it is difficult to distinguish the two species (Rafi et al., 2013). The cp sequence is significant for studying phylogenetic relationships, genetic diversity and species identification (Daniell et al., 2016; Teske et al., 2020). Thus, we analyzed the cp genomes of two *Zingiber* species. Similar to other studies, the complete cp genomes of *Z. corallinum* and *Z. montanum* composed of a single circular molecule with obvious quadripartite structure (Zhou et al., 2017; Liang et al., 2020). According to cp genome features analysis of *Z. corallinum* and *Z. montanum*, there was no difference in the number and location of genes except the length of LSC and IR.

In the present study, we found that more codons showed the codon usage bias (RSCU > 1) in the cp genes of two *Zingiber* species. This phenomenon of high codon preference is very common in the chloroplast genome of higher plants (Kim and Lee, 2004). Some studies believe that the high RSCU value may be determined by the structure of amino acids or the function of the peptide to avoid errors in transcription (Banerjee and Ghosh, 2006). Furthermore, other than leucine (UUG), amino acid codons in two *Zingiber* species cp genomes of preferentially ended with A or U (RSCU > 1), and codons ending with A or U accounted for 71.1%. The start codon AUG and UGG encoding methionine and tryptophan were not biased (RSCU = 1). These results are similar to those observed for Dracaena species and *Zingiber officinale* (Cui et al., 2019; Zhang et al., 2019). SSRs are usually used as an important molecular marker for species identification and diversity analysis (Powell et al., 1995). In this paper, 110 SSRs were identified from the cp genomes of two *Zingiber* species, and the most abundant repeat type was AT/TA, followed by A/T mononucleotide repeat. Previous studies found that cp genome SSRs always consists of short poly-A or poly-T repeats, and most plants rarely contain tandem G or C repeats (Li et al., 2021). At present, SSR markers have been widely used in the fields of species identification, genetic diversity, population structure evaluation, comparative genomics, and marker-assisted selection breeding (Zhang et al., 2019; Liang et al., 2020). Although the codon number and frequency, SSRs location and types of *Z. corallinum* and *Z. montanum* in this study were exactly the same, these identified repetitive sequences will still provide valuable resources for species identification and population studies of other *Zingiber* species.

We found the two cp genomes had no variable sites and *Z. corallinum* had deletion fragments 526 bp relative to *Z. montanum* by used DnaSP software. Meanwhile, we downloaded the *Z. montanum* (MK262727) sequence published by NCBI (Li et al., 2020a), and compared the sequence homology of *Z. corallinum*, *Z. montanum*, and *Z. montanum* (MK262727). The comparison showed that the differences among the three sequences were very small, and their nucleotide variability (Pi) values were 0.0033 and 0.0044. Therefore, although there were many studies using the cp genome to screen highly variable regions as molecular markers for molecular identification (Kelly et al., 2011; Liu et al., 2015), this method was not applicable to the two *Zingiber* species in this study.

We speculated the phylogenetic relationships of the two *Zingiber* species within the Zingiberaceae using complete cp genomes. Both the MP and NJ phylogenetic trees indicated that the two genera *Alpinia* and *Amomum* as sister groups into a branch were strongly supported (support values ≥ 95%), the results were similar to complete cp genomes of three medicinal *Alpinia* species research (Li et al., 2020b). In general, our research results were basically consistent with the morphological classification and further confirmed the phylogenetic relationship of Zingiberaceae (Li et al., 2020b). Meanwhile, two phylogeny trees both showed that the sequence difference between *Z. corallinum*, *Z. montanum*, and *Z. montanum* (MK262727) were very infinitely small, but the NJ tree confirmed the *Z. montanum* and *Z. montanum* (MK262727) into a small branch (support values = 100%), and MP tree confirmed the *Z. montanum*, *Z. montanum* (MK262727), and *Z. corallinum* into a paratactic branch (support values = 100%). To sum up, the cp genome could not guarantee accurate identification.

Based on the results of cp genomes, we can see that the difference between *Z. corallinum* and *Z. montanum* was very small. Thus, cp genomes could not provide valuable discriminatory power to distinguish the two *Zingiber* species from each other. In recent years, untargeted metabolomics have been demonstrated to be powerful tools for herbal medicine authentication (Park et al., 2019; Wang et al., 2019; Rastogi et al., 2020; Sánchez et al., 2020). As an important part of systematic biology, the application research of untargeted metabolomics in medicinal plants has continued to expand, and its role in the identification of TCMs has become increasingly prominent. Endara et al. (2018) proposed the concept of “Chemocoding” using untargeted metabolomics in combination with multivariate analysis and proved that it can provide an additional identification tool for species identification comparing with traditional morphological and molecular-based taxonomic identification methods. In addition to species identification, there are also some reports on metabolomics studies in different medicinal parts, different populations, and authenticity (Black et al., 2016; Lee et al., 2017; Shao et al., 2019).

Here, we focused on developing chemical markers to distinguish *Z. corallinum* and *Z. montanum* based on untargeted metabolomics. In the metabolic profiling analysis, the two species showed no qualitative differences in major volatile compounds, but there were obvious quantitative differences in major volatile compounds. The results of PCA and OPLS-DA indicated that substantial differences in the content of metabolites existed in *Z. corallinum* and *Z. montanum*. Obviously, metabolic profile was more powerful in distinguishing *Zingiber* species than cp genomes. This supports the argument that metabolic markers can be used effectively to identify relationships between plant species. The reason may be that the levels of metabolites are hyper-sensitive to environmental factors and growth stages compared with DNA markers (Duan et al., 2011). It turned out that metabolic profiling was the efficient tool to successfully solve the identification debate about TCMs (Duan et al., 2011; Endara et al., 2018). Moreover, the candidate biomarkers for the discrimination of *Zingiber* materials may provide a basis to reveal the different metabolism in the rhizomes of these two *Zingiber* species in further analysis.

## Conclusion

This study contributes an integrated strategy to discriminate closely related TCM materials by using chloroplast genomics and untargeted metabolomics as a case of *Z. corallinum* and *Z. montanum*. The cp genome-based fingerprinting indicated that the two *Zingiber* species are closely related, which is not enough to distinguish *Z. corallinum* and *Z. montanum*. Thus, a GC-MS-based untargeted metabolic analytical methodology was proposed for rapid analysis of a large number of *Zingiber* samples. The strategy used here enabled the two *Zingiber* species to be discriminated. This discrimination was achieved by detecting nine potential biomarkers which were found to be specific for species. This predictive approach was confirmed to unequivocally classify species of the unknown samples. Our results suggested that the metabolic profiling could be easily used to discriminate the two species. In the future study, the untargeted metabolomics approach is expected to play an important role in the classification and discrimination of TCMs.

## Data Availability Statement

The original contributions presented in the study are publicly available. This data can be found here: Two complete cp genome sequences of *Z. corallinum* and *Z. montanum* were submitted to GenBank database, and their accession numbers were MW801385 and MW801386, respectively.

## Author Contributions

CW and ZZ designed and conducted the research. ZZ, YZ, and MS collected samples and determined the species. CW contributed to the untargeted metabolomic analysis. YZ performed the chloroplast genome sequence and analysis on the data. CW and YZ wrote the manuscript. LH revised the manuscript. All authors have read and approved the final manuscript.

## Conflict of Interest

The authors declare that the research was conducted in the absence of any commercial or financial relationships that could be construed as a potential conflict of interest.

## Publisher’s Note

All claims expressed in this article are solely those of the authors and do not necessarily represent those of their affiliated organizations, or those of the publisher, the editors and the reviewers. Any product that may be evaluated in this article, or claim that may be made by its manufacturer, is not guaranteed or endorsed by the publisher.

## Footnotes

1
http://dogma.ccbb.utexas.edu/

2
http://pgrc.ipk-gatersleben.de/misa/
