# Supplementary material for: Authentication of Zingiber Species Based on Analysis of Metabolite Profiles
Source: Front Plant Sci. 2021 Nov 22;12:705446. doi: 10.3389/fpls.2021.705446 (PMC8647842; doi:10.3389/fpls.2021.705446)
Supplement: Supplementary file 1 [file Data_Sheet_1.docx]

Supplementary Material

# Supplementary Figures and Tables

## Supplementary Figures


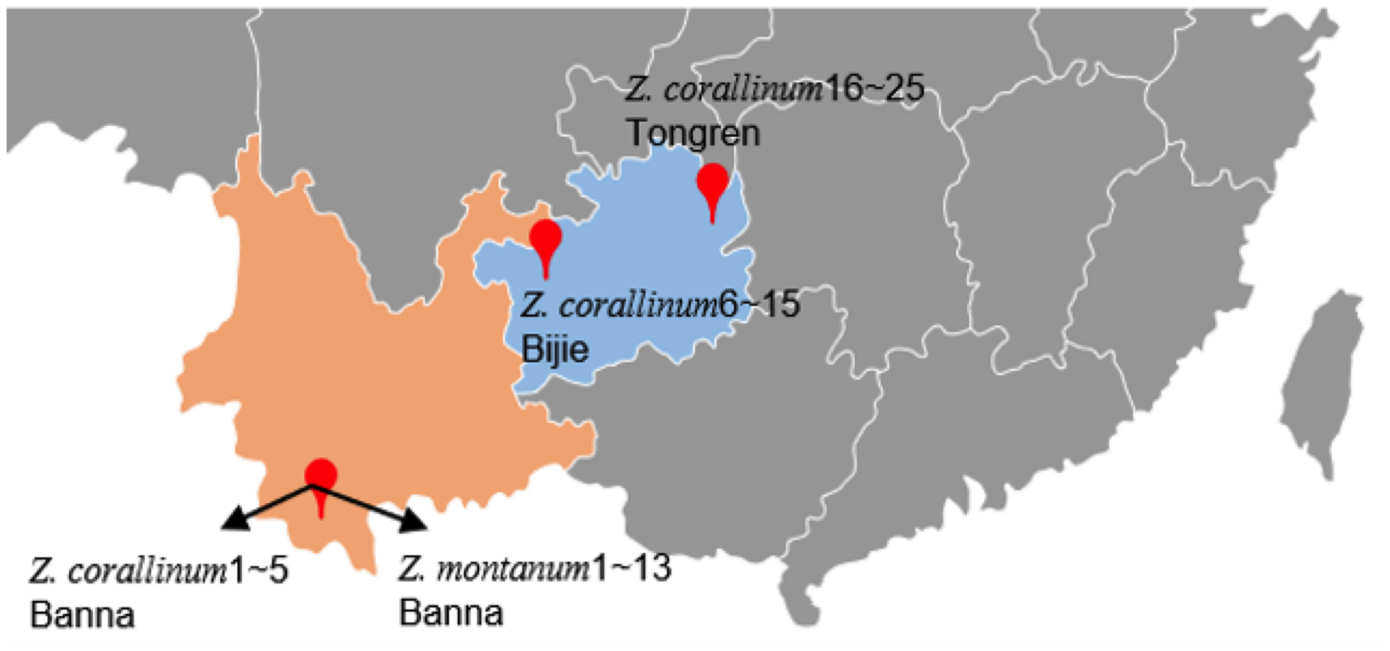


**Supplementary Figure 1.** *Zingiber* species used in this study and collection areas are indicated on the map.

**
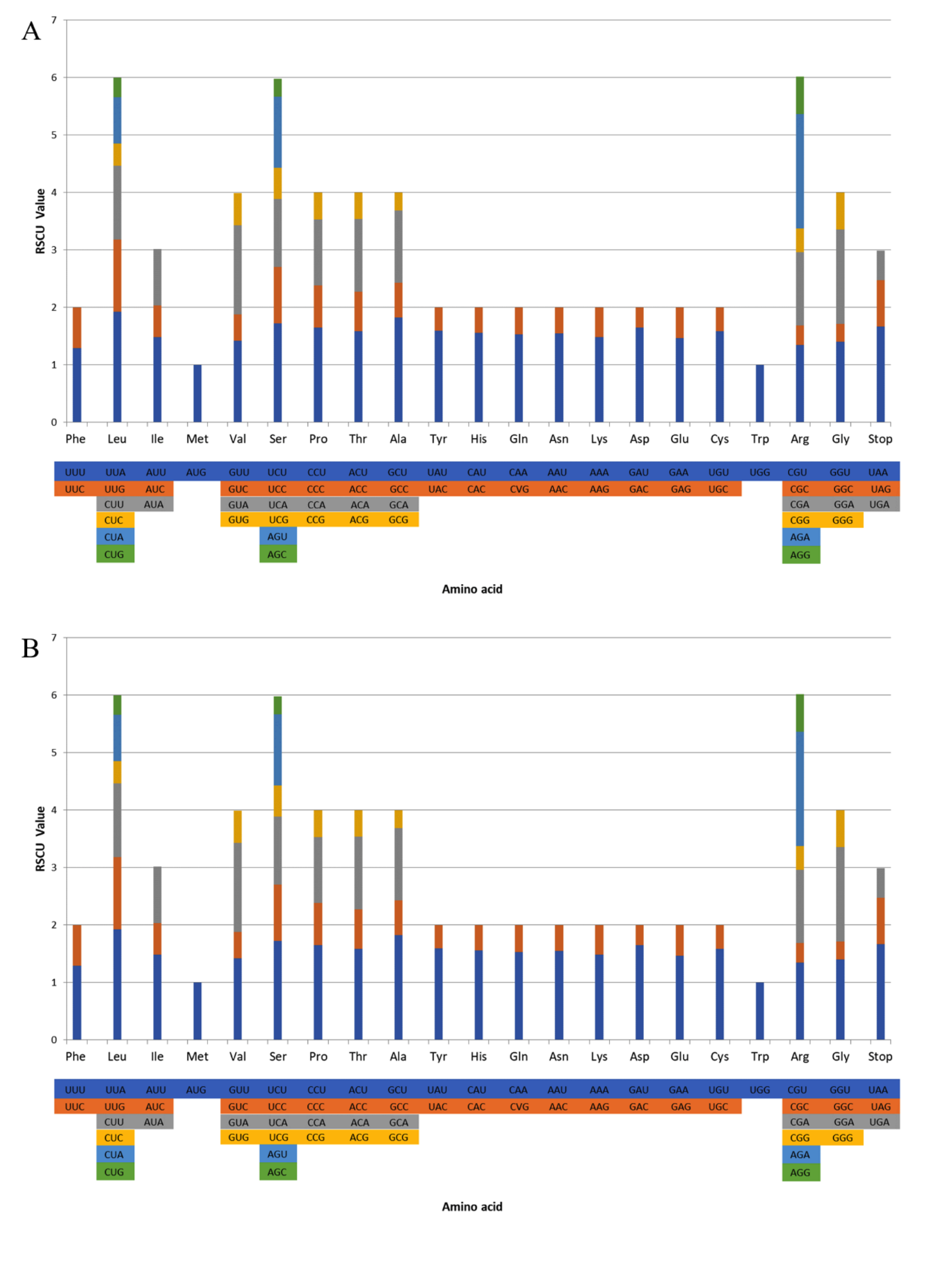
**

**Supplementary Figure 2.** Codon content of all protein‐coding genes in the *Z. corallinum* (A) and *Z. montanum* (B) chloroplast genome.

**
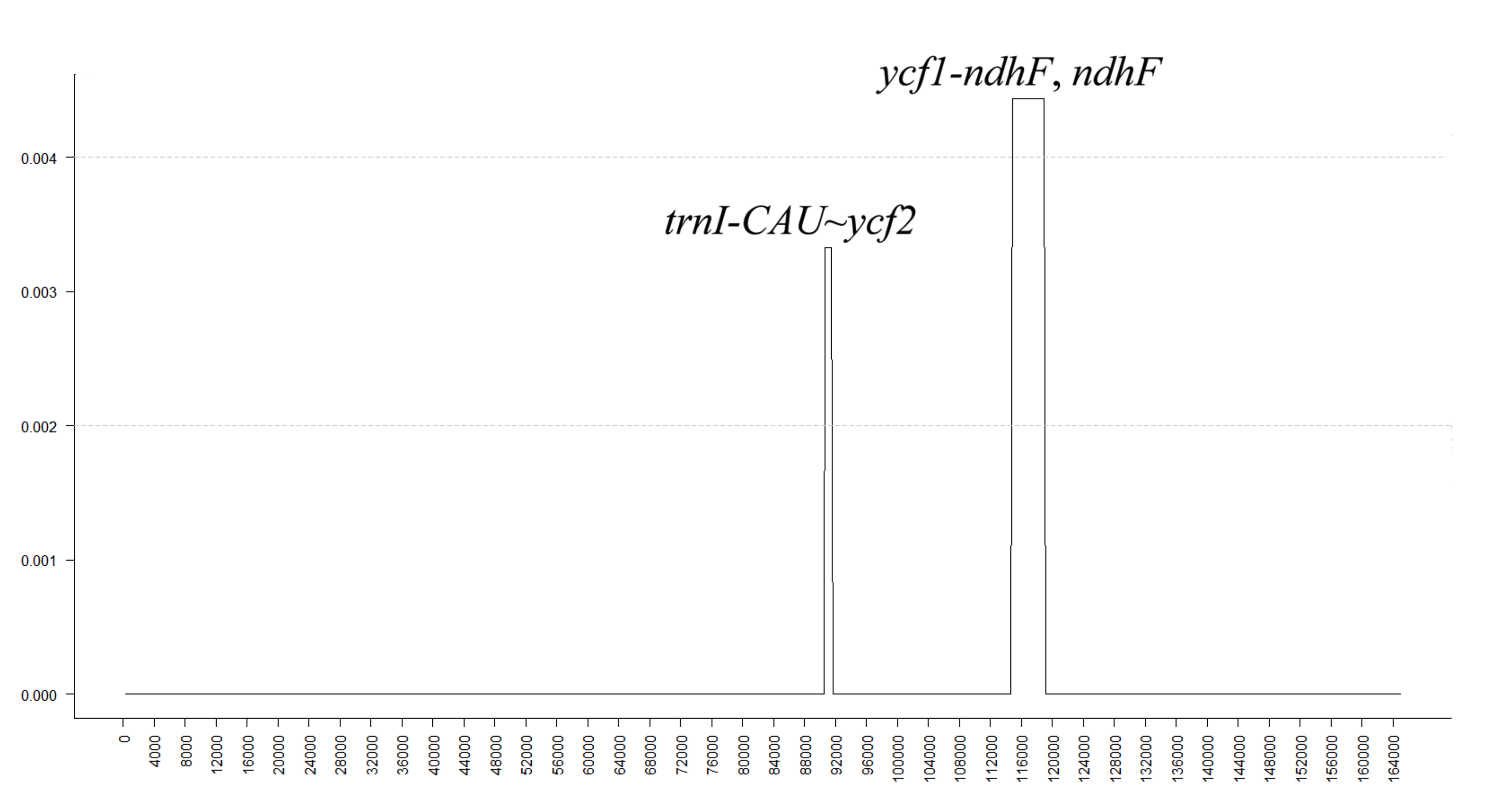
**

**Supplementary Figure 3.** The variable regions in the cp genome of *Z. corallinum* and *Z. montanum*.

**
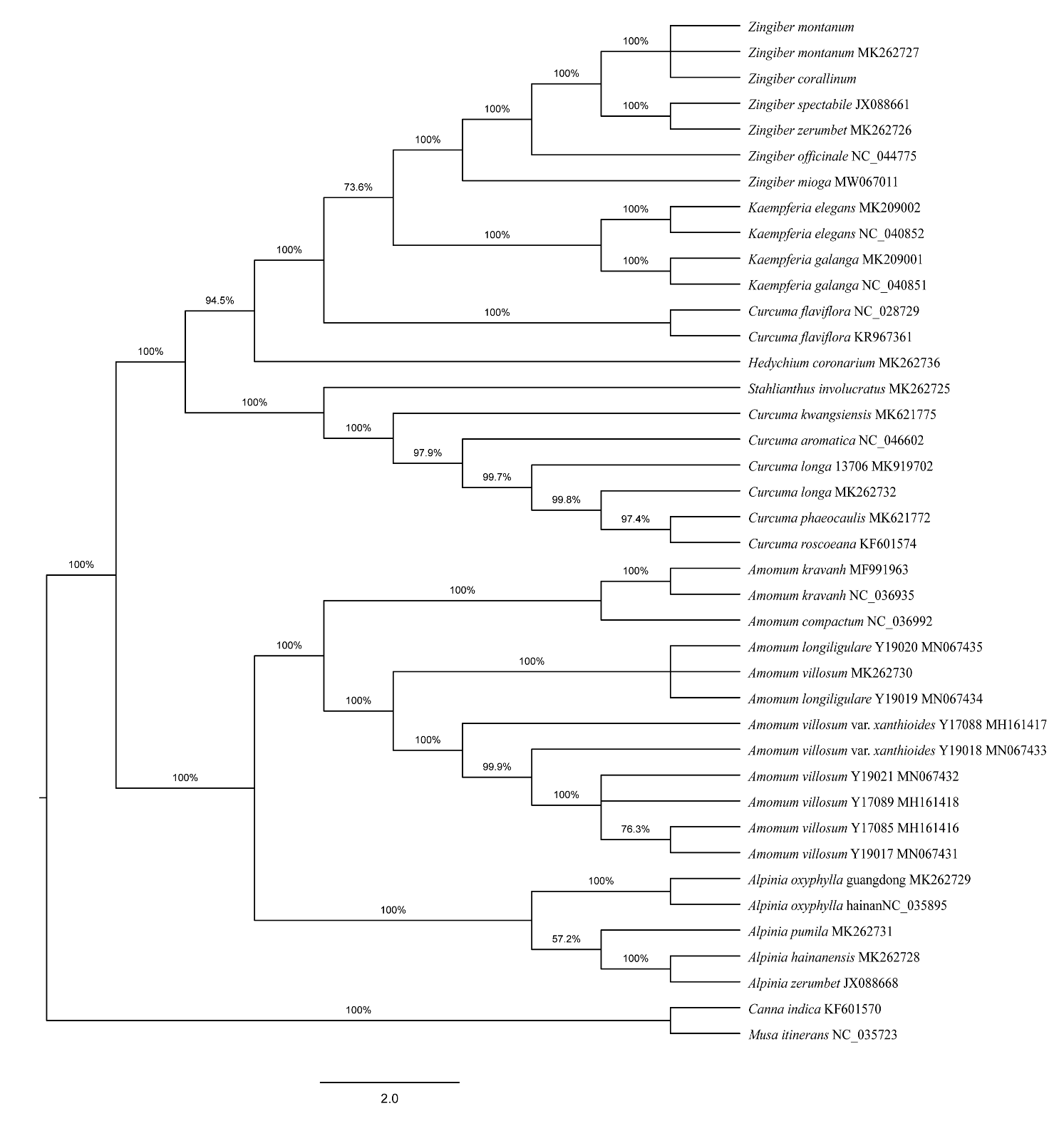
**

**Supplementary Figure 4.** Phylogenetic tree constructed using Maximum Parsimony (MP) based on complete cp genomes of 36 Zingiberaceae species. Numbers above the branches are the bootstrap support values.


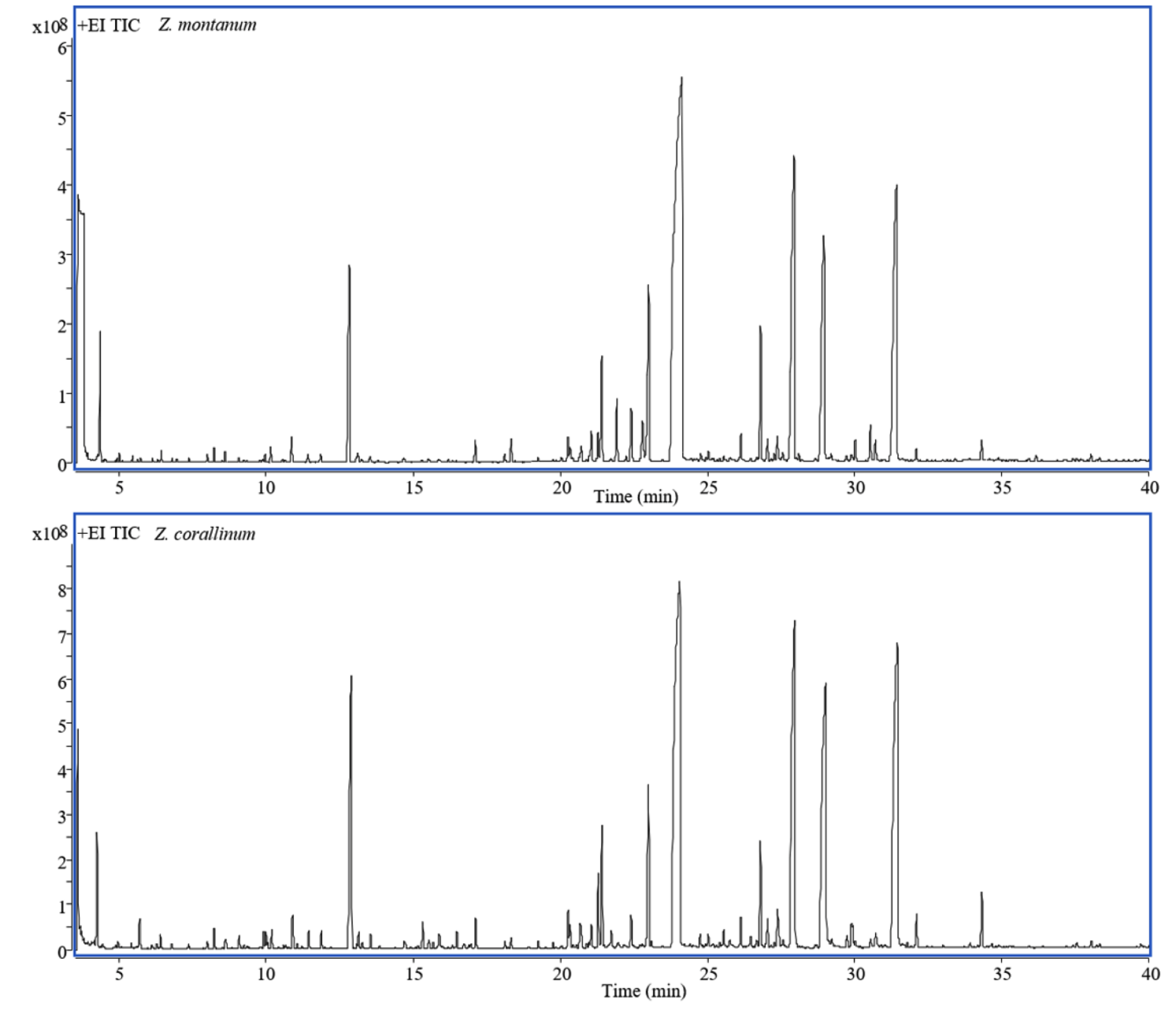


**Supplementary Figure 5.** GC-MS total ion (TIC) chromatograms of two *Zingiber* species.


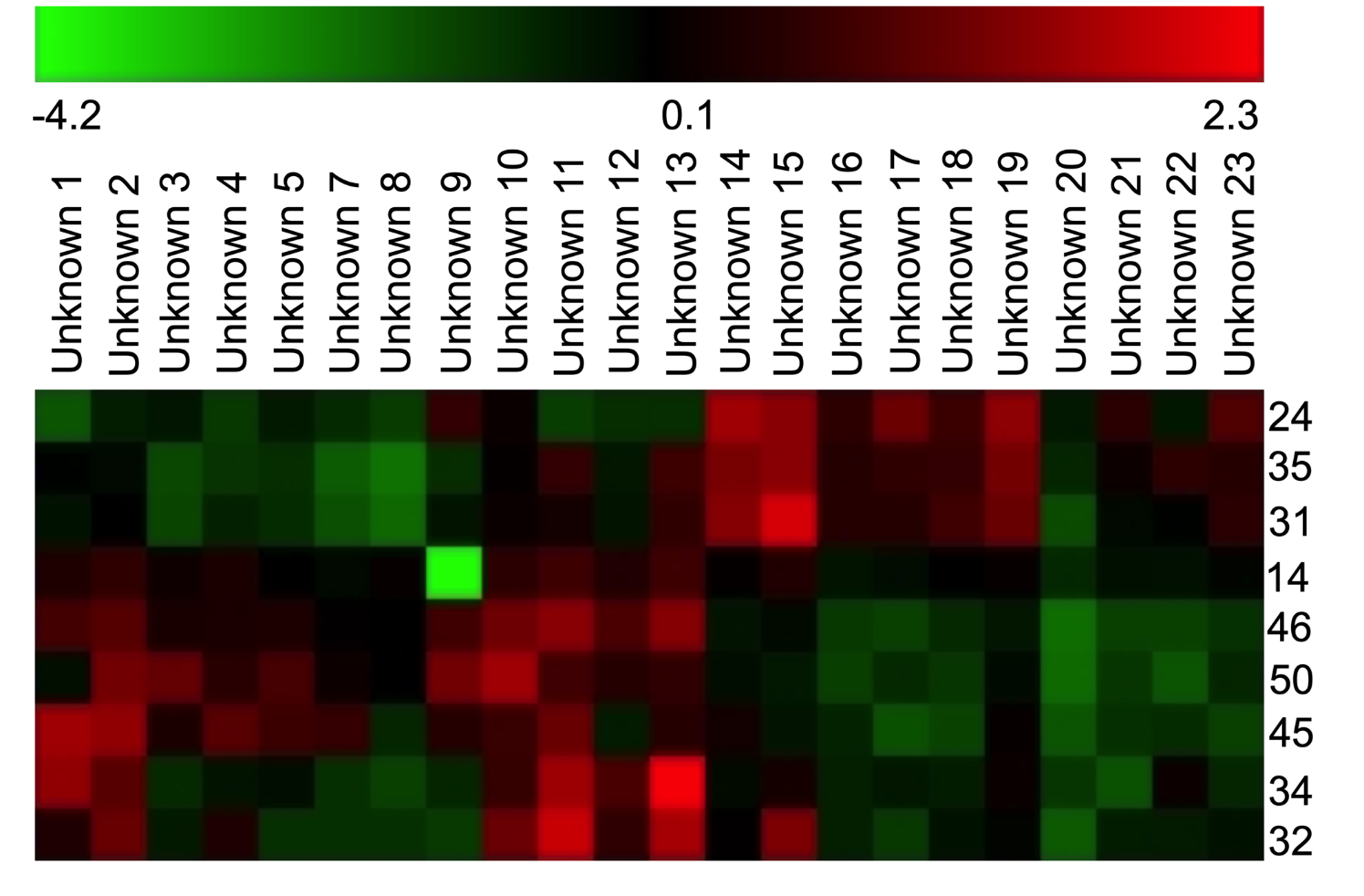


**Supplementary Figure 6.** Heatmap of identified distinguished metabolites by the unknown samples.

## Supplementary Tables

**Supplementary Table 1** List of *Zingiber* species used in the study.

| Species | Sample | Geographical origin | Geographical location |
| --- | --- | --- | --- |
| *Zingiber corallinum*  (*Z. corallinum*) | *Z. corallinum* 1* | Banna Pharmaceutical, Yunnan Province | E:100°52'12" N:22°01'36" |
|  | *Z. corallinum* 2 | Banna Pharmaceutical, Yunnan Province | E:100°52'12" N:22°01'36" |
|  | *Z. corallinum* 3 | Banna Pharmaceutical, Yunnan Province | E:100°52'12" N:22°01'36" |
|  | *Z. corallinum* 4 | Banna Pharmaceutical, Yunnan Province | E:100°52'12" N:22°01'36" |
|  | *Z. corallinum* 5 | Banna Pharmaceutical, Yunnan Province | E:100°52'12" N:22°01'36" |
|  | *Z. corallinum* 6 | Bijie City, Guizhou Province | E:105°35'24" N: 27°18'36" |
|  | *Z. corallinum* 7 | Bijie City, Guizhou Province | E:105°35'24" N: 27°18'36" |
|  | *Z. corallinum* 8 | Bijie City, Guizhou Province | E:105°40'12" N: 27°25'12" |
|  | *Z. corallinum* 9 | Bijie City, Guizhou Province | E:105°40'12" N: 27°25'12" |
|  | *Z. corallinum* 10 | Bijie City, Guizhou Province | E:105°40'12" N: 27°25'12" |
|  | *Z. corallinum* 11 | Bijie City, Guizhou Province | E:105°25'12" N: 27°12'36" |
|  | *Z. corallinum* 12 | Bijie City, Guizhou Province | E:105°25'12" N: 27°12'36" |
|  | *Z. corallinum* 13 | Bijie City, Guizhou Province | E:105°25'12" N: 27°12'36" |
|  | *Z. corallinum* 14 | Bijie City, Guizhou Province | E:105°25'12" N: 27°12'36" |
|  | *Z. corallinum* 15 | Bijie City, Guizhou Province | E:105°25'12" N: 27°12'36" |
|  | *Z. corallinum* 16 | Tongren City, Guizhou Province | E:109°25'48" N: 28°06'36" |
|  | *Z. corallinum* 17 | Tongren City, Guizhou Province | E:109°25'48" N: 28°06'36" |
|  | *Z. corallinum* 18 | Tongren City, Guizhou Province | E:109°25'53" N: 28°06'87" |
|  | *Z. corallinum* 19 | Tongren City, Guizhou Province | E:109°25'55" N: 28°06'87" |
|  | *Z. corallinum* 20 | Tongren City, Guizhou Province | E:109°24'58" N: 28°04'22" |
|  | *Z. corallinum* 21 | Tongren City, Guizhou Province | E:109°24'58" N: 28°04'22" |
|  | *Z. corallinum* 22 | Tongren City, Guizhou Province | E:109°29'24" N: 27°48'36" |
|  | *Z. corallinum* 23 | Tongren City, Guizhou Province | E:109°29'59" N: 27°48'02" |
|  | *Z. corallinum* 24 | Tongren City, Guizhou Province | E:109°22'12" N: 27°39'36" |
|  | *Z. corallinum* 25 | Tongren City, Guizhou Province | E:109°23'42" N: 28°33'11" |
| *Zingiber montanum*  (*Z. montanum*) | *Z. montanum* 1* | Banna Pharmaceutical, Yunnan Province | E:100°52'12" N:22°01'36" |
|  | *Z. montanum* 2 | Banna Pharmaceutical, Yunnan Province | E:100°52'12" N:22°01'36" |
|  | *Z. montanum* 3 | Banna Pharmaceutical, Yunnan Province | E:100°52'12" N:22°01'36" |
|  | *Z. montanum* 4 | Banna Pharmaceutical, Yunnan Province | E:100°49'48" N:22°01'12" |
|  | *Z. montanum* 5 | Banna Pharmaceutical, Yunnan Province | E:100°49'48" N:22°01'12" |
|  | *Z. montanum* 6 | Banna Pharmaceutical, Yunnan Province | E:100°49'48" N:22°01'12" |
|  | *Z. montanum* 7 | Banna Pharmaceutical, Yunnan Province | E:100°49'48" N:22°01'12" |
|  | *Z. montanum* 8 | Banna Pharmaceutical, Yunnan Province | E:101°18'48" N:21°59'01" |
|  | *Z. montanum* 9 | Banna Pharmaceutical, Yunnan Province | E:101°18'48" N:21°59'01" |
|  | *Z. montanum* 10 | Banna Pharmaceutical, Yunnan Province | E:101°18'48" N:21°59'01" |
|  | *Z. montanum* 11 | Banna Pharmaceutical, Yunnan Province | E:100°49'89" N:22°01'19" |
|  | *Z. montanum* 12 | Banna Pharmaceutical, Yunnan Province | E:100°49'89" N:22°01'19" |
|  | *Z. montanum* 13 | Banna Pharmaceutical, Yunnan Province | E:100°50'02" N:22°01'22" |

*: Plant materials used for complete chloroplast genome sequencing.

**Supplementary Table 2** Base composition of the *Z. corallinum* and *Z. montanum* chloroplast genome.

|  |  | *Z. corallinum* | | | | | *Z. montanum* | | | | |
| --- | --- | --- | --- | --- | --- | --- | --- | --- | --- | --- | --- |
|  |  | T(U) (%) | C (%) | A (%) | G (%) | Length (bp) | T(U) (%) | C (%) | A (%) | G (%) | Length (bp) |
| Total |  | 32.4 | 18.3 | 31.7 | 17.6 | 160957 | 32.5 | 18.2 | 31.7 | 17.6 | 161483 |
| IRa |  | 29.4 | 21.7 | 28.8 | 20.2 | 26817 | 29.5 | 21.5 | 28.8 | 20.2 | 27035 |
| IRb |  | 28.8 | 20.2 | 29.4 | 21.7 | 26817 | 28.8 | 20.2 | 29.5 | 21.5 | 27035 |
| LSC |  | 33.8 | 17.2 | 32.6 | 16.4 | 87835 | 33.8 | 17.2 | 32.6 | 16.4 | 87925 |
| SSC |  | 35.7 | 15.5 | 35.0 | 13.8 | 19488 | 35.7 | 15.5 | 35.0 | 13.8 | 19488 |
| CDS |  | 31.7 | 17.1 | 31.2 | 19.9 | 79464 | 31.7 | 17.1 | 31.2 | 19.9 | 79464 |
|  | 1st position | 24.0 | 18.2 | 31.2 | 26.5 | 26488 | 24.0 | 18.2 | 31.2 | 26.5 | 26488 |
|  | 2nd position | 32.6 | 20.0 | 29.9 | 17.5 | 26488 | 32.6 | 20.0 | 29.9 | 17.5 | 26488 |
|  | 3rd position | 38.6 | 13.2 | 32.5 | 15.7 | 26488 | 38.6 | 13.2 | 32.5 | 15.7 | 26488 |

**Supplementary Table 3** Length and location of *Z. corallinum* and *Z. montanum* genes.

| GeneName | *Z. corallinum* | *Z. montanum* | Structure |
| --- | --- | --- | --- |
| accD | 1554 | 1554 | LSC |
| atpA | 1524 | 1524 | LSC |
| atpB | 1488 | 1488 | LSC |
| atpE | 408 | 408 | LSC |
| atpF | 570 | 570 | LSC |
| atpH | 246 | 246 | LSC |
| atpI | 744 | 744 | LSC |
| ccsA | 990 | 990 | SSC |
| cemA | 690 | 690 | LSC |
| clpP | 615 | 615 | LSC |
| infA | 234 | 234 | LSC |
| matK | 1548 | 1548 | LSC |
| ndhA | 1080 | 1080 | SSC |
| ndhB | 1566 | 1566 | IRa |
| ndhB | 1566 | 1566 | IRb |
| ndhC | 363 | 363 | LSC |
| ndhD | 1506 | 1506 | SSC |
| ndhE | 306 | 306 | SSC |
| ndhF | 2217 | 2217 | SSC |
| ndhG | 531 | 531 | SSC |
| ndhH | 1182 | 1182 | SSC |
| ndhI | 543 | 543 | SSC |
| ndhJ | 480 | 480 | LSC |
| ndhK | 753 | 753 | LSC |
| petA | 963 | 963 | LSC |
| petB | 648 | 648 | LSC |
| petD | 489 | 489 | LSC |
| petG | 114 | 114 | LSC |
| petL | 96 | 96 | LSC |
| petN | 90 | 90 | LSC |
| psaA | 2253 | 2253 | LSC |
| psaB | 2205 | 2205 | LSC |
| psaC | 246 | 246 | SSC |
| psaI | 111 | 111 | LSC |
| psaJ | 129 | 129 | LSC |
| psbA | 1062 | 1062 | LSC |
| psbB | 1527 | 1527 | LSC |
| psbC | 1422 | 1422 | LSC |
| psbD | 1062 | 1062 | LSC |
| psbE | 252 | 252 | LSC |
| psbF | 120 | 120 | LSC |
| psbH | 222 | 222 | LSC |
| psbI | 111 | 111 | LSC |
| psbJ | 123 | 123 | LSC |
| psbK | 186 | 186 | LSC |
| psbL | 117 | 117 | LSC |
| psbM | 105 | 105 | LSC |
| psbN | 132 | 132 | LSC |
| psbT | 108 | 108 | LSC |
| psbZ | 189 | 189 | LSC |
| rbcL | 1443 | 1443 | LSC |
| rpl14 | 369 | 369 | LSC |
| rpl16 | 411 | 411 | LSC |
| rpl20 | 429 | 429 | LSC |
| rpl22 | 330 | 330 | LSC |
| rpl23 | 282 | 282 | IRa |
| rpl23 | 282 | 282 | IRb |
| rpl2 | 828 | 828 | IRa |
| rpl2 | 828 | 828 | IRb |
| rpl32 | 174 | 174 | SSC |
| rpl33 | 201 | 201 | LSC |
| rpl36 | 114 | 114 | LSC |
| rpoA | 1020 | 1020 | LSC |
| rpoB | 3228 | 3228 | LSC |
| rpoC1 | 2064 | 2064 | LSC |
| rpoC2 | 4140 | 4140 | LSC |
| rps11 | 417 | 417 | LSC |
| rps12 | 372 | 372 | IRa |
| rps12 | 372 | 372 | IRb |
| rps14 | 303 | 303 | LSC |
| rps15 | 273 | 273 | SSC |
| rps16 | 252 | 252 | LSC |
| rps18 | 315 | 315 | LSC |
| rps19 | 285 | 285 | Ira |
| rps19 | 285 | 285 | Irb |
| rps2 | 711 | 711 | LSC |
| rps3 | 660 | 660 | LSC |
| rps4 | 609 | 609 | LSC |
| rps7 | 468 | 468 | IRa |
| rps7 | 468 | 468 | IRb |
| rps8 | 399 | 399 | LSC |
| rrn16 | 1491 | 1491 | IRa |
| rrn16 | 1491 | 1491 | IRb |
| rrn23 | 2808 | 2808 | IRa |
| rrn23 | 2808 | 2808 | IRb |
| rrn4.5 | 103 | 103 | IRa |
| rrn4.5 | 103 | 103 | IRb |
| rrn5 | 121 | 121 | IRa |
| rrn5 | 121 | 121 | IRb |
| trnA-UGC | 73 | 73 | IRa |
| trnA-UGC | 73 | 73 | IRb |
| trnC-GCA | 71 | 71 | LSC |
| trnD-GUC | 74 | 74 | LSC |
| trnE-UUC | 73 | 73 | LSC |
| trnF-GAA | 73 | 73 | LSC |
| trnfM-CAU | 74 | 74 | LSC |
| trnG-GCC | 71 | 71 | LSC |
| trnG-UCC | 71 | 71 | LSC |
| trnH-GUG | 75 | 75 | IRa |
| trnH-GUG | 75 | 75 | IRb |
| trnI-CAU | 74 | 74 | IRa |
| trnI-CAU | 74 | 74 | IRb |
| trnI-GAU | 77 | 77 | IRa |
| trnI-GAU | 77 | 77 | IRb |
| trnK-UUU | 72 | 72 | LSC |
| trnL-CAA | 81 | 81 | IRa |
| trnL-CAA | 81 | 81 | IRb |
| trnL-UAA | 85 | 85 | LSC |
| trnL-UAG | 80 | 80 | SSC |
| trnM-CAU | 72 | 72 | LSC |
| trnN-GUU | 72 | 72 | IRa |
| trnN-GUU | 72 | 72 | IRb |
| trnP-UGG | 74 | 74 | LSC |
| trnQ-UUG | 72 | 72 | LSC |
| trnR-ACG | 74 | 74 | IRa |
| trnR-ACG | 74 | 74 | IRb |
| trnR-UCU | 72 | 72 | LSC |
| trnS-GCU | 88 | 88 | LSC |
| trnS-GGA | 87 | 87 | LSC |
| trnS-UGA | 92 | 92 | LSC |
| trnT-GGU | 72 | 72 | LSC |
| trnT-UGU | 73 | 73 | LSC |
| trnV-GAC | 72 | 72 | IRa |
| trnV-GAC | 72 | 72 | IRb |
| trnV-UAC | 75 | 75 | LSC |
| trnW-CCA | 74 | 74 | LSC |
| trnY-GUA | 84 | 84 | LSC |
| ycf1 | 308 | 308 | IRb |
| ycf1 | 5508 | 5508 | SSC |
| ycf2 | 6885 | 6885 | IRa |
| ycf2 | 6885 | 6885 | IRb |
| ycf3 | 513 | 513 | LSC |
| ycf4 | 555 | 555 | LSC |

**Supplementary Table 4** Relative synonymous codon usage in *Z. corallinum* and *Z. montanum*.

| *Z. corallinum*: Average# codons=26488 | | | | | | | | | | | |
| --- | --- | --- | --- | --- | --- | --- | --- | --- | --- | --- | --- |
| Codon | Count | RSCU | Codon | Count | RSCU | Codon | Count | RSCU | Codon | Count | RSCU |
| UUU(F) | 969 | 1.29 | UCU(S) | 594 | 1.72 | UAU(Y) | 806 | 1.59 | UGU(C) | 241 | 1.58 |
| UUC(F) | 529 | 0.71 | UCC(S) | 336 | 0.98 | UAC(Y) | 211 | 0.41 | UGC(C) | 65 | 0.42 |
| UUA(L) | 883 | 1.92 | UCA(S) | 411 | 1.19 | UAA (*) | 48 | 1.67 | UGA (*) | 15 | 0.52 |
| UUG(L) | 577 | 1.26 | UCG(S) | 185 | 0.54 | UAG (*) | 23 | 0.8 | UGG(W) | 455 | 1 |
| CUU(L) | 590 | 1.28 | CCU(P) | 443 | 1.65 | CAU(H) | 500 | 1.56 | CGU(R) | 356 | 1.34 |
| CUC(L) | 180 | 0.39 | CCC(P) | 197 | 0.73 | CAC(H) | 141 | 0.44 | CGC(R) | 90 | 0.34 |
| CUA(L) | 371 | 0.81 | CCA(P) | 310 | 1.15 | CAA(Q) | 708 | 1.53 | CGA(R) | 340 | 1.28 |
| CUG(L) | 154 | 0.34 | CCG(P) | 127 | 0.47 | CAG(Q) | 217 | 0.47 | CGG(R) | 108 | 0.41 |
| AUU(I) | 1152 | 1.48 | ACU(T) | 528 | 1.58 | AAU(N) | 998 | 1.55 | AGU(S) | 435 | 1.26 |
| AUC(I) | 425 | 0.55 | ACC(T) | 230 | 0.69 | AAC(N) | 292 | 0.45 | AGC(S) | 106 | 0.31 |
| AUA(I) | 761 | 0.98 | ACA(T) | 423 | 1.27 | AAA(K) | 1075 | 1.48 | AGA(R) | 530 | 1.99 |
| AUG(M) | 618 | 1 | ACG(T) | 155 | 0.46 | AAG(K) | 375 | 0.52 | AGG(R) | 174 | 0.65 |
| GUU(V) | 508 | 1.42 | GCU(A) | 614 | 1.82 | GAU(D) | 889 | 1.65 | GGU(G) | 605 | 1.4 |
| GUC(V) | 165 | 0.46 | GCC(A) | 205 | 0.61 | GAC(D) | 191 | 0.35 | GGC(G) | 134 | 0.31 |
| GUA(V) | 552 | 1.55 | GCA(A) | 421 | 1.25 | GAA(E) | 1057 | 1.46 | GGA(G) | 711 | 1.64 |
| GUG(V) | 201 | 0.56 | GCG(A) | 107 | 0.32 | GAG(E) | 391 | 0.54 | GGG(G) | 280 | 0.65 |
| *Z. montanum*: Average# codons=26488 | | | | | | | | | | | |
| Codon | Count | RSCU | Codon | Count | RSCU | Codon | Count | RSCU | Codon | Count | RSCU |
| UUU(F) | 969 | 1.29 | UCU(S) | 594 | 1.72 | UAU(Y) | 806 | 1.59 | UGU(C) | 241 | 1.58 |
| UUC(F) | 529 | 0.71 | UCC(S) | 336 | 0.98 | UAC(Y) | 211 | 0.41 | UGC(C) | 65 | 0.42 |
| UUA(L) | 883 | 1.92 | UCA(S) | 411 | 1.19 | UAA (*) | 48 | 1.67 | UGA (*) | 15 | 0.52 |
| UUG(L) | 577 | 1.26 | UCG(S) | 185 | 0.54 | UAG (*) | 23 | 0.8 | UGG(W) | 455 | 1 |
| CUU(L) | 590 | 1.28 | CCU(P) | 443 | 1.65 | CAU(H) | 500 | 1.56 | CGU(R) | 356 | 1.34 |
| CUC(L) | 180 | 0.39 | CCC(P) | 197 | 0.73 | CAC(H) | 141 | 0.44 | CGC(R) | 90 | 0.34 |
| CUA(L) | 371 | 0.81 | CCA(P) | 310 | 1.15 | CAA(Q) | 708 | 1.53 | CGA(R) | 340 | 1.28 |
| CUG(L) | 154 | 0.34 | CCG(P) | 127 | 0.47 | CAG(Q) | 217 | 0.47 | CGG(R) | 108 | 0.41 |
| AUU(I) | 1152 | 1.48 | ACU(T) | 528 | 1.58 | AAU(N) | 998 | 1.55 | AGU(S) | 435 | 1.26 |
| AUC(I) | 425 | 0.55 | ACC(T) | 230 | 0.69 | AAC(N) | 292 | 0.45 | AGC(S) | 106 | 0.31 |
| AUA(I) | 761 | 0.98 | ACA(T) | 423 | 1.27 | AAA(K) | 1075 | 1.48 | AGA(R) | 530 | 1.99 |
| AUG(M) | 618 | 1 | ACG(T) | 155 | 0.46 | AAG(K) | 375 | 0.52 | AGG(R) | 174 | 0.65 |
| GUU(V) | 508 | 1.42 | GCU(A) | 614 | 1.82 | GAU(D) | 889 | 1.65 | GGU(G) | 605 | 1.4 |
| GUC(V) | 165 | 0.46 | GCC(A) | 205 | 0.61 | GAC(D) | 191 | 0.35 | GGC(G) | 134 | 0.31 |
| GUA(V) | 552 | 1.55 | GCA(A) | 421 | 1.25 | GAA(E) | 1057 | 1.46 | GGA(G) | 711 | 1.64 |
| GUG(V) | 201 | 0.56 | GCG(A) | 107 | 0.32 | GAG(E) | 391 | 0.54 | GGG(G) | 280 | 0.65 |
